# Case Report: Combined Liver-Kidney Transplantation to Correct a Mutation in Complement Factor B in an Atypical Hemolytic Uremic Syndrome Patient

**DOI:** 10.3389/fimmu.2021.751093

**Published:** 2021-10-14

**Authors:** Margarita López-Trascasa, Ángel Alonso-Melgar, Marta Melgosa-Hijosa, Laura Espinosa-Román, María Dolores Lledín-Barbancho, Eugenia García-Fernández, Santiago Rodríguez de Córdoba, Pilar Sánchez-Corral

**Affiliations:** ^1^ Departamento de Medicina, Universidad Autónoma de Madrid, Madrid, Spain; ^2^ Complement Research Group, Hospital La Paz Institute for Health Research (IdiPAZ), La Paz University Hospital, Madrid, Spain; ^3^ Pediatric Nephrology Service, La Paz University Hospital, Madrid, Spain; ^4^ Pediatric Hepatology Service, La Paz University Hospital, Madrid, Spain; ^5^ Pathology Department, La Paz University Hospital, Madrid, Spain; ^6^ Department of Molecular Biomedicine, Centro de Investigaciones Biológicas (CSIC), Madrid, Spain; ^7^ Center for Biomedical Network Research on Rare Diseases (CIBERER), Madrid, Spain

**Keywords:** atypical hemolytic uremic syndrome, complement, factor B, combined liver-kidney transplantation, rare diseases

## Abstract

Pathogenic gain-of-function variants in complement Factor B were identified as causative of atypical Hemolytic Uremic syndrome (aHUS) in 2007. These mutations generate a reduction on the plasma levels of complement C3. A four-month-old boy was diagnosed with hypocomplementemic aHUS in May 2000, and he suffered seven recurrences during the following three years. He developed a severe hypertension which required 6 anti-hypertensive drugs and presented acrocyanosis and several confusional episodes. Plasma infusion or exchange, and immunosuppressive treatments did not improve the clinical evolution, and the patient developed end-stage renal disease at the age of 3 years. Hypertension and vascular symptoms persisted while he was on peritoneal dialysis or hemodialysis, as well as after bilateral nephrectomy. C3 levels remained low, while C4 levels were normal. In 2005, a heterozygous gain-of-function mutation in Factor B (K323E) was found. A combined liver and kidney transplantation (CLKT) was performed in March 2009, since there was not any therapy for complement inhibition in these patients. Kidney and liver functions normalized in the first two weeks, and the C3/C4 ratio immediately after transplantation, indicating that the C3 activation has been corrected. After remaining stable for 4 years, the patient suffered a B-cell non-Hodgkin lymphoma that was cured by chemotherapy and reduction of immunosuppressive drugs. Signs of liver rejection with cholangitis were observed a few months later, and a second liver graft was done 11 years after the CLKT. One year later, the patient maintains normal kidney and liver functions, also C3 and C4 levels are within the normal range. The 12-year follow-up of the patient reveals that, in spite of severe complications, CLKT was an acceptable therapeutic option for this aHUS patient.

## Introduction

Hemolytic uremic syndrome (HUS) is characterized by the triad of hemolytic anemia, acute kidney failure, and thrombocytopenia. Most cases are children with infections by *Escherichia Coli* 0157:H7, *Shigella or Salmonella*, and generally evolve satisfactorily in a few weeks. Less frequently, HUS develops in the absence of a clear etiologic agent and has a worse evolution; these cases are denominated as atypical Hemolytic Uremic Syndrome (aHUS), which is very uncommon and is considered an ultrarare disease (1). Around 50-60% of aHUS patients have pathogenic variants in the complement genes *CFH, MCP, CFI*, *C3* and *CFB*, or circulating anti-factor H autoantibodies (2). Moreover, pathogenic variants outside the complement system have been described in thrombomodulin, DGKE and plasminogen genes.

Mutations in complement *CFH, CFI, C3* or *CFB* generate anomalies in the corresponding proteins, which are mainly produced in the liver and secreted into plasma; these anomalies persist after kidney transplantation, a reason that can explain the high level of disease recurrence in transplanted patients. Most of these complement pathogenic variants were described on the beginning of this century, when the complement inhibitor Eculizumab was not yet accessible, and combined liver-kidney transplantation was considered a therapy to cure HUS in patients with mutations in *CFH* or *CFI* (3).

*CFB* mutations were first described in aHUS patients in 2007 (4). In this paper, two different mutations in complement factor B (FB) were identified. A pedigree with several members affected shared mutation F286L, while the second mutation, K323E, was a “*de novo*” mutation in an unrelated patient. The two mutations generated hyperfunctional FB proteins which gave rise to a more active C3bBb convertase and an important C3 consumption in plasma, which was particularly evident in the patient carrying the K323E mutation. The follow-up of one patient from the F286L pedigree has been recently described (5); this patient underwent a kidney transplant without Eculizumab treatment in 2011 and has not suffered HUS relapses.

The patient carrying the K323E mutation underwent a combined liver kidney transplantation (CLKT) in March 2009; a short summary of the evolution during the first three months post-transplantation has been previously reported (6). In this work, we fully describe the clinical evolution, transplantation history and complement levels of this patient, throughout his 20 years of life. The patient presented HUS when he was 4-months old, suffered multiple recurrences and incidents for 8 years, and received a combined liver kidney transplantation (CLKT) at the age of 9 years. This CLKT restored complement levels as well as kidney and liver functions after the transplant surgery. The patient had several transplant related complications such as a B-cell non-Hodgkin lymphoma, and a chronic liver rejection that required a second liver graft 11 years after CLKT. In spite of these complications, CLKT was at that time the only opportunity for improving the critical situation of the patient, and the function of the kidney graft has remained normal all the time.

## Case Description, Diagnostic Assessment, Therapeutic Intervention, Follow-Up and Outcomes

A 4-month-old male was diagnosed with HUS and treated with peritoneal dialysis from the 5^th^ day of disease onset. The patient had a bad evolution, with renal failure, anemia, severe hypertension, and peritonitis by *Staphylococcus Epidermidis*, and after 20 days he suffered a second HUS episode and was transferred to our Hospital. At that moment, in addition to altered hematologic parameters, complement analysis indicated very low C3 levels (35,4 mg/dL; normal range: 77-135) with normal C4 (43,8 mg/dL; normal range: 14-60); Factor H, Factor I, and MCP levels were on the normal range. Hematological parameters and glomerular filtration normalized after 1 month, but hypertension remained partially uncontrolled and hemolysis markers such as haptoglobin were persistently low. After 6 months, the patient suffered a third episode with severe hypertension which required as many as six hypotensive drugs. Moreover, he endured two episodes of acute pulmonary edema with respiratory distress caused by hypertensive crisis. He developed a severe anemia that required 17 erythrocyte concentrates, mild thrombocytopenia, proteinuria in the nephrotic range, and cardiac insufficiency. He also suffered acute renal failure which required diuretic and hemodialysis support. A renal biopsy performed during this episode indicated thrombotic microangiopathy, with major vascular involvement and mild glomerular damage (**Supplementary Figure 1**).

**Figure 1:**
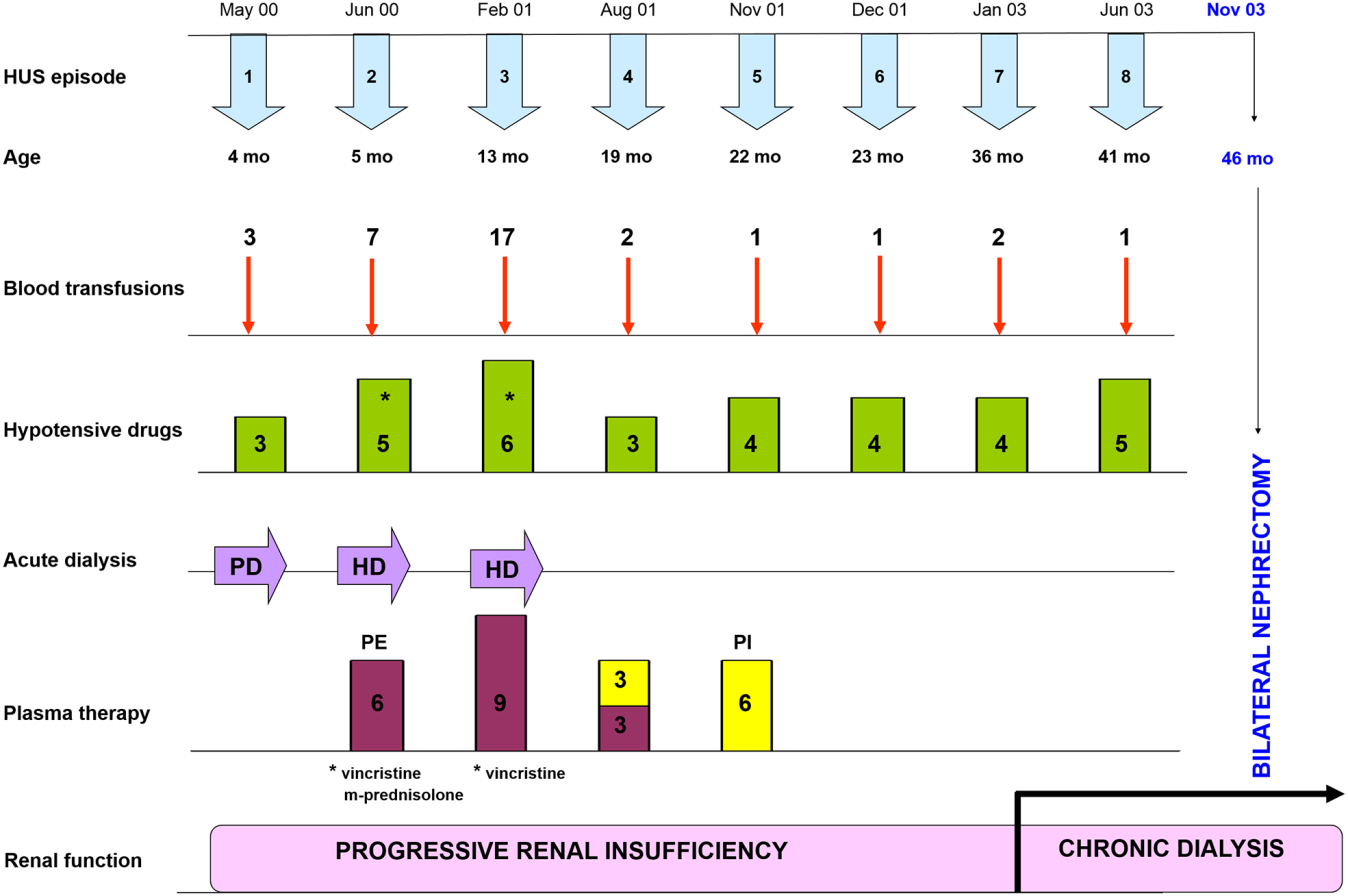
Clinical evolution since HUS onset to bilateral nephrectomy. Timeline of HUS episodes, treatments and renal outcome along the first four years of life of the patient. Supportive treatments included blood transfusions and plasma therapy, as well as different combinations of hypotensive drugs (Hydralazine, Atenolol, Furosemide, Nifedipine, Minoxidil and Enalapril); immunosuppressive drugs (Vincristine and M-Prednisolone) were used occasionally. The patient entered into chronic dialysis at the age of 36 months, and at the age of 46 months both kidneys had to be removed to avoid further HUS episodes. PD, Peritoneal Dialysis; HD, Hemodialysis; PE, Plasma Exchange; PI, Plasma Infusion.

The patient suffered 5 subsequent HUS episodes; treatment with immunosuppressive drugs (vincristine and prednisone) and several plasma exchanges courses were tried without success. Uncontrolled hypertension with hemodynamic instability required prolonged hospitalizations. He underwent chronic dialysis at the age of three years, and 10 months later both kidneys were removed in an attempt to avoid the severe complications associated to HUS episodes (**Figure 1**). Nonetheless, the hypertension continued after bilateral nephrectomy, and the patient had several episodes of acrocyanosis on all the fingers of the lower extremities (Raynaud’s phenomenon) because of systemic microangiopathy (**Supplementary Figure 2**).

Although hematological parameters normalized after bilateral nephrectomy, the clinical course of the patient under chronic hemodialysis continued to be torpid, with severe hypertension with cardiac repercussion, stroke-like episodes not clearly explained, and bad tolerance to hemodialysis sessions. All the time, plasma levels of complement C3 were low, while levels of C4 and C5 were normal. When he was 5 years-old, a pathogenic gain-of-function variant in complement Factor B (FB) was identified (4); genetic studies also showed the presence of the two aHUS-risk haplotypes *CFH(H3*) y *MCPggaac* in heterozygosis. This molecular diagnosis opened the possibility of a combined liver kidney transplantation (CLKT), since by that time complement inhibition with Eculizumab was not available. While waiting for CLKT, the patient continued with a regimen of hemodialysis and plasma infusions.

**Figure 2:**
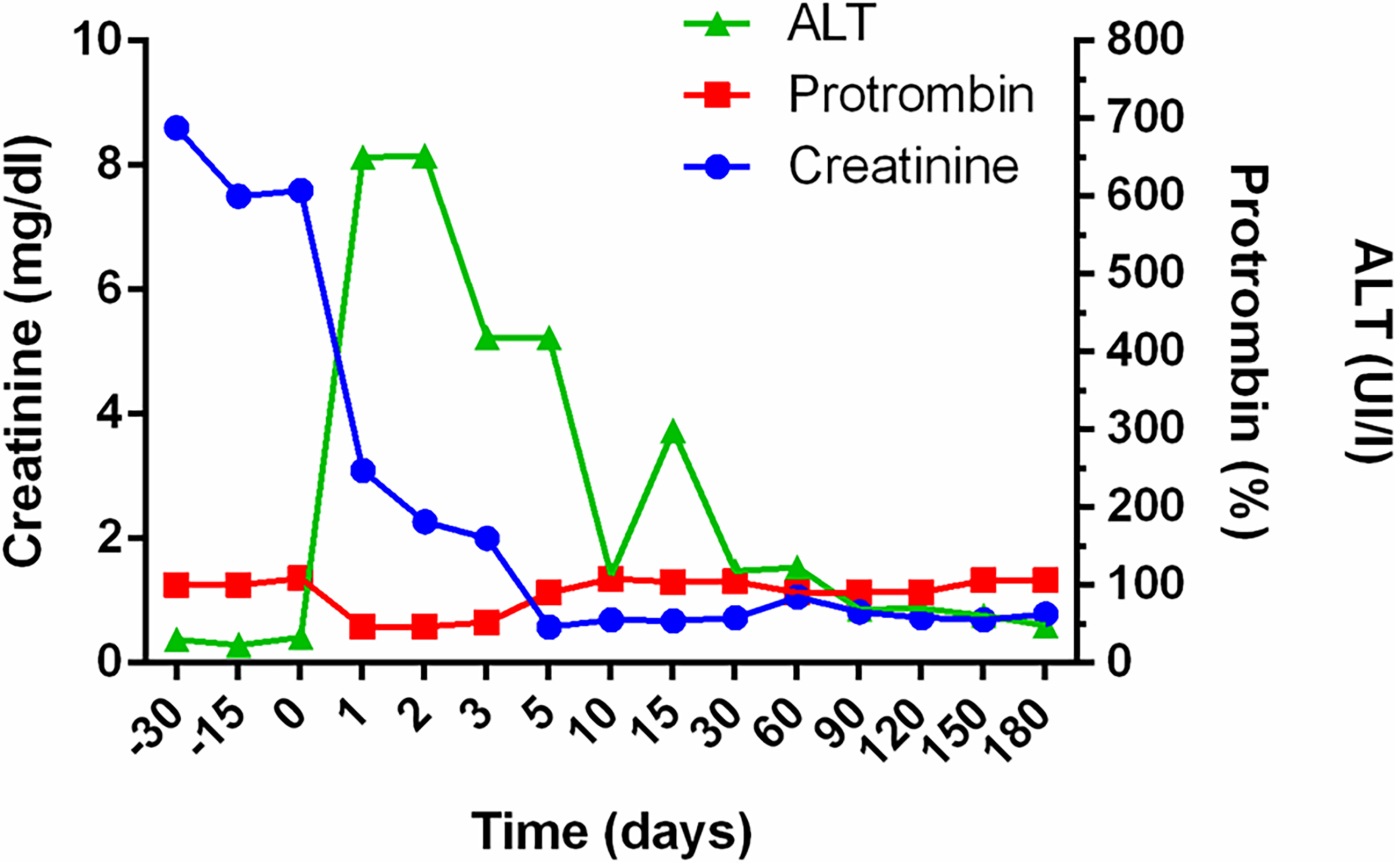
Evolution of renal and hepatic function after combined liver-kidney transplantation. Follow up of Creatinine, Prothrombin and Alaninaminotransferase (ALT) levels in the patient before and after transplantation. Creatinine levels dropped immediately after transplant surgery, reaching normal levels 5 days later. The hepatic function normalized during the second week.

**Figure 3:**
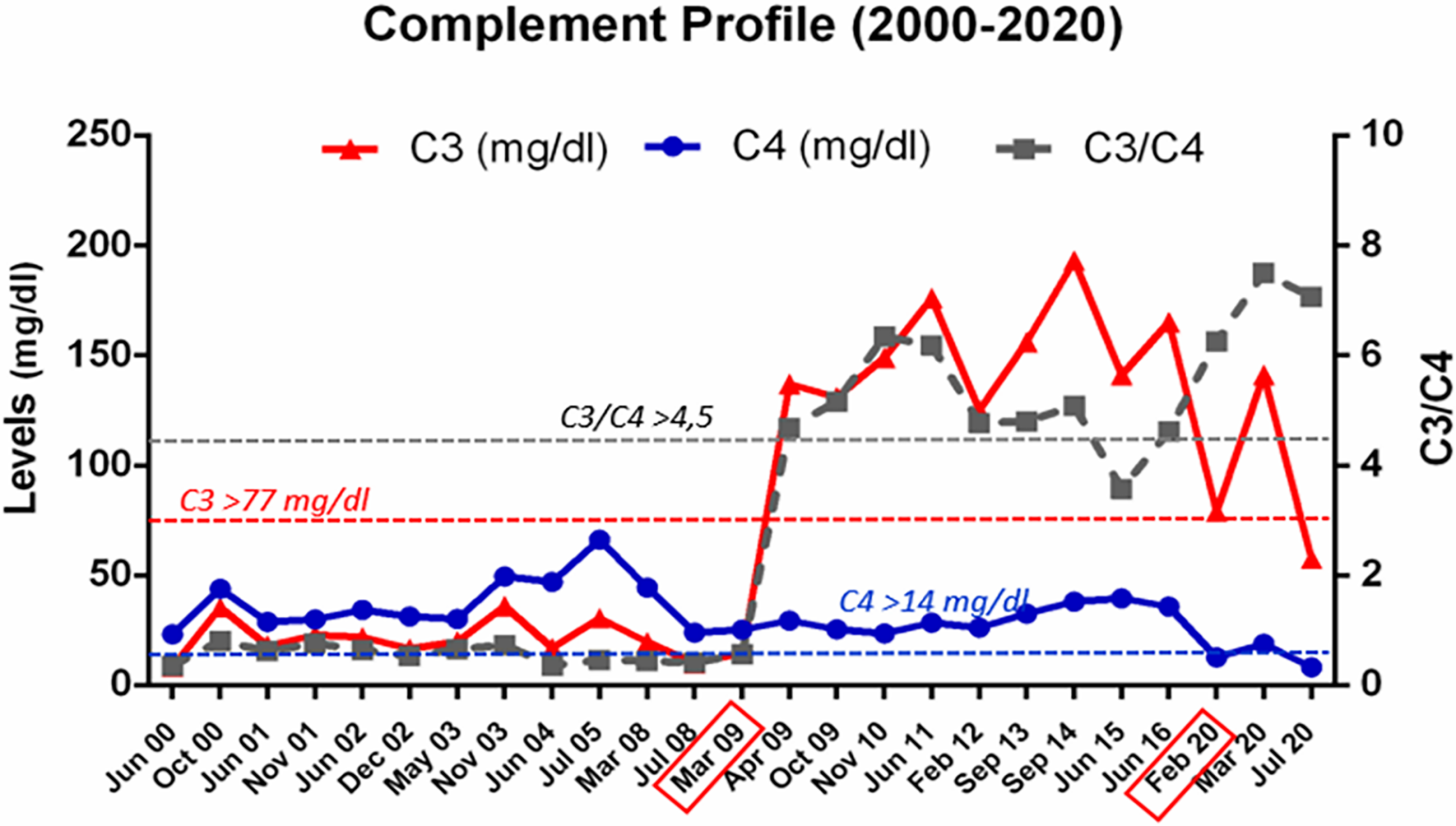
Complement profile from 2000 to 2020. Evolution of C3 and C4 levels, and of the C3/C4 ratio, in plasma samples from patient HUS21 since HUS onset in 2000, at the age of 4 months. The horizontal dashed lines indicate the lower limit of normal values. The boxes in the X axis mark the dates of the liver-kidney transplantation (March 2009), and of the second liver transplant (February 2020).

At the age of 9 years, the patient received a CLKT from an 8-year-old child. Immediately before the surgery, he was treated with intensive plasma exchange to ensure removal of his circulating FB; two other plasma exchange sessions were performed the third- and fourth-days post-transplantation, coinciding with mild anemization without signs of hemolysis. Systemic heparinization was used as part of our liver transplant protocol. Normal kidney and liver functions were evident 2 weeks post-transplantation (**Figure 2**), and plasma levels of complement C3 reached normal values for the first time in the patient’s life (**Figure 3**). The patient remained in hospital for 30 days. Suspicion of hepatic acute rejection in early postoperative period was treated with a pulse of metilprednisolone, with good response; histopathology of the biopsy discarded the rejection. The patient continued stable for 4 years under immunosuppressive treatment (induction with Basiliximab, and maintenance with corticosteroids, tacrolimus and mycophenolate mofetil).

On September 2013, the patient was diagnosed with a diffuse large B cell non-Hodgkin’s lymphoma stage 3, with infiltration in scalp and skull, and was treated with chemotherapy (R-CHOP/R-COPDAM) and rituximab. At the beginning of the treatment, he suffered a septic shock and endured at Intensive Care Unit for 9 days. The infectious episode induced coagulopathy and multiorgan failure (with hepatic, kidney, and cardiac involvement) with good outcome. After 3 months, the chemotherapy was ended with evident signs of remission. All along the chemotherapy treatment, tacrolimus and mycophenolate mofetil were stopped and only corticosteroids were maintained.

On February 2014, he suffered from a liver graft dysfunction with a light increase of transaminases without ultrasonographic signs of dilated biliary tract. He received intravenous antibiotics and ursodeoxycholic acid by suspicion for cholangitis. Liver biopsy showed moderate periportal fibrosis, but no histological features of chronic rejection. Subsequently to liver rejection in 2014, the patient developed high levels of *de novo* donor-specific antibodies (DSA) against DQ9 (MFI: 21705). These DSA were maintained with a similar MFI (between 21000 and 32000) at least until February 2020, just before the second liver graft; moreover, serological viral studies were negative. Sirolimus was included in the immunosuppressive treatment, and the corticosteroids and ursodeoxycholic acid doses were increased. Following several cholangitis and infectious episodes, in 2018 a second liver biopsy showed ductal proliferation with inflammation and branding fibrosis. Diagnosis of chronic liver rejection was established, and tacrolimus and mycophenolate mofetil were reintroduced. Percutaneous transhepatic cholangiography showed multiple biliary structures of the biliary tree without obstruction in the anastomotic site. Several biliary dilatations and external biliary drainage were performed, and finally, a biliary stent was positioned. However, the patient continued suffering cholangitis episodes by multidrug-resistant bacteria, requiring frequent hospitalization. During all this period, the glomerular filtration rate decreased during the acute infection episodes but normalized afterwards, with no signs of kidney rejection. On February 2020, the patient received a second cadaveric liver graft. By that moment, creatinine-based glomerular filtration rate was normal (> 90 ml/min/1.73 m2).

Currently, the patient has a good clinical condition, although he displays a low height for its expected genetic height (height: 158.5 cm (-3.18SD); weight: 56.2Kg (-1.79 SD); data related to Spanish population). He receives immunosuppressive treatment with corticosteroids, tacrolimus and mycophenolate mofetil, with maintained hepatic function after his second liver graft: AST 34 UI/L (<40); ALT 48 UI/L (<35); GGT 30 UI/L (<73); total bilirubin 0.79 mg/dL (0.30-1.20), albumin 4.3 g/dL (2.9-5.2). Kidney function is also good, with a normal GFR calculated both by Creatinine and by Cystatin C (creatinine 1 mg/dl (0.70-1.30); GFR calculated by CKD-EPI >90 ml/min/1.73 m2 (N >75); Cystatin C 1.20 mg/L (0.64-1.23); GFR calculated by CKD-EPI Cyst C 72 mL/min/1.73m2 (N>60); protein/creatinine ratio 0.08 (N<0.2).

**Figure 4:**
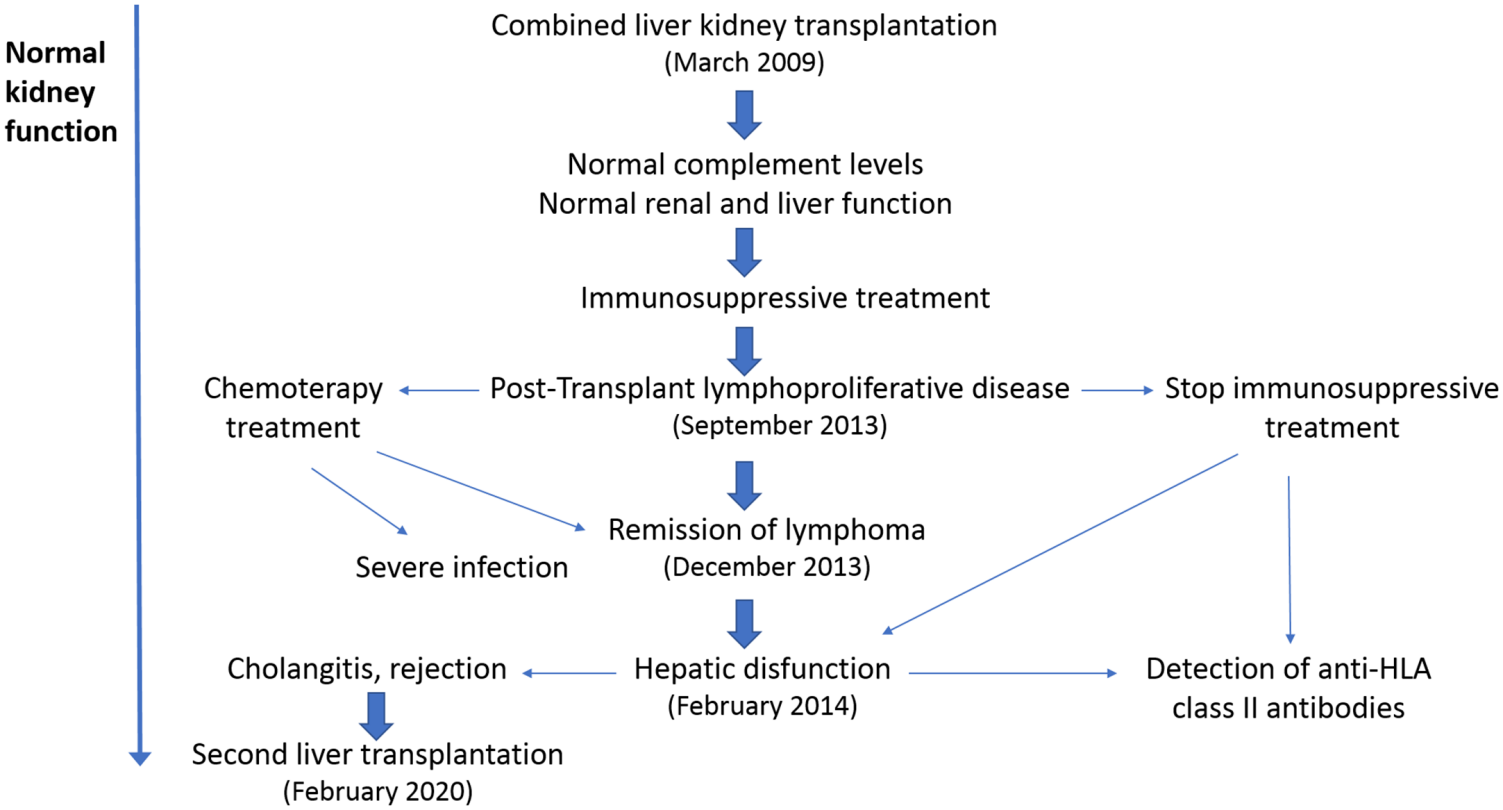
Post-transplant clinical evolution. The main clinical episodes after combined liver-kidney transplantation (CLKT) are indicated. The first complication, 4 years after transplantation, was a lymphoproliferative disease, which was treated with chemotherapy and reduction/abrogation of the immunosuppressive regimen. Immunosuppression was reintroduced 5 months later, after a hepatic failure and detection of high levels of anti-HLA-II antibodies. Taking into account the bad evolution of the liver graft, the patient underwent a second liver transplant in 2020, which remains functional until now. In spite of these findings, the renal function has remained stable since the CLKT in 2009.

The main clinical and treatment events after CLKT are shown in **Figure 4**; as shown in **Figure 3**, plasma levels of C3, C4, and the C3/C4 ratio, have always remained within the normal range. Now, the patient is under the supervision on a different hospital (adult section) for the hepatic and kidney post-transplantation follow-up.

## Discussion, Take-Away Lessons

In the last 20 years, there has been an important improvement in the comprehension of the molecular bases underlying atypical Hemolytic Uremic Syndrome. The complement system was initially considered an important mediator of the endothelial damage characteristic of this rare disease, and the subsequent identification of pathogenic variants and/or polymorphisms in one or more complement genes further strengthen its role as disease causative (2). In the aHUS patient described here, the identification of a gain-of-function mutation in complement Factor B explained the high and constitutive consumption of complement C3 in the patient´s plasma.

The fact that the liver is the main source of plasma Factor B (7) anticipated HUS recurrence after an isolated kidney transplantation, thus hampering this possibility for our patient. By that time, several aHUS patients with mutations in Factor H, most of them children, underwent CLKT. Although initial experiences had a fatal outcome (8–10), the introduction of pre-emptive plasmapheresis with plasma exchange proved to be very successful (11–13). At the light of these experiences, and in the absence of other therapies, it was decided that the CKLT was the only curative option for our patient with the Factor B mutation. This decision was adopted after a consensus meeting between nephrologists, hepatologists, surgeons, and immunologists from Hospital La Paz, and complement researchers involved in the case. In spite of its risks, the intervention was accepted by the patient’s parents, and it could be done in 2009, upon the availability of liver and kidney grafts from a donor of the same age that the patient. The CLKT allowed immediate restoration of the renal function in the patient, who remained without problems under immunosuppressive treatment for four years.

A B-cell lymphoma was the first complication of the patient. The B-cell lymphoma could be related with the immunosuppressive treatment, but the association of immunosuppression with subsequent complications after CLKT is only partially understood (14–16). The risk of tumor appearance induced by the immunosuppression treatment increases with postransplantation time. The overall incidence of tumors in the pediatric age after a solid organ transplantation is around 6%, with values of 7% in liver and around 3% in renal grafts. There are few data on combined transplantation, but tumor incidence seems to be like that of single liver transplantation, around 8% (17). These percentages raise when the follow-up is prolonged into adulthood. In all solid organ transplantation, between 80 and 90% of the tumors are of lymphoid origin. In addition, in our patient, the immunosuppression received prior to transplantation could increase the tumor risk.

The immunosuppression regimes in CLKT and isolated kidney transplantation are very similar; moreover, in CLKT the liver graft confers immunoprotection to the kidney graft. This effect was first observed in experimental animals (18) and later on in patients (19). The presence of DSA was considered a predisposition factor to graft rejection in isolated kidney transplantation (20, 21). However, in the case of CLKT, the liver graft protects the kidney from the hyperacute reaction, and it also decreases the acute cellular response (22). In a more recent cooperative study, the authors evidenced that the presence of DSA antibodies in CLKT rises the mortality, but they did not find a clear relationship with the kidney and liver graft evolution (23).

Cholangitis associated to chronic liver rejection was an additional and severe complication in the patient, with a 6-year evolution that conducted to a second liver graft in 2020 that remains functional. Although the patient suffered several complications along the post-transplant period, in 2009 CLKT was the only therapeutic approach, since complement inhibitors such as Eculizumab were not available. The combined transplantation allowed the patient to leave dialysis and to improve his social and academic life; currently, he is enrolled on a university degree. Further complications were solved in a tertiary hospital, and the patient continues under immunosuppressive therapy with adequate follow-up.

To correct the complement dysregulation and the very low C3 levels caused by the gain-of-function mutation in Factor B was an important consideration when discussing the therapeutic options for our patient. Our conclusion was that, although liver transplantation would add additional risks to kidney transplantation, the CLKT was the only available option to completely normalize complement activation. Nowadays, our patient would have likely been approached with an isolated kidney transplantation and the use of Eculizumab. However, during the last 15 years it has been an extraordinary progress in the development of drugs inhibiting different levels of the complement cascade, providing novel therapeutic options to aHUS patients. In this changing scenario, a next generation of complement inhibitors acting upstream of C5 activation would soon be available for the treatment of complement dysregulation disorders. One of these novel drugs, which may be better and more appropriate than Eculizumab for some patients, is Pegcetacoplan (24, 25), a PEGylated pentadecapeptide that binds to C3 and C3b, and regulates the activation of C3 and the activity of the AP C3 convertase. Pegcetacoplan has been recently approved by the FDA for the treatment of adults with paroxysmal nocturnal hemoglobinuria (26).

CLKT is still nowadays a valid therapeutic option for aHUS patients. Thus, the experts recommend not to completely discard CLKT (which could be the only option in countries or situations where Eculizumab is not available), and to discuss all the options with patients and families (27). CLKT in aHUS patients with complement mutations has been extensively reviewed by Zuber et al. in 2013 (28), and by Saland et al. in 2014 (29); these papers included 16 cases with *CFH* mutations (14 successful), one with *C3* mutation (unsuccessful), one with *CFB* mutation (this patient), 2 cases with a hybrid *CFH::CFHR1* gene (one successful), and an additional case as personal communication (28).

Functional characterization of the Factor B K323E variant purified from the patient’s plasma showed increased resistance of the C3 convertase to dissociation by the complement regulators Factor H and DAF (4). The gain-of-function consequences of the K323E variant were confirmed in another study where Factor B pathogenic variants identified in other aHUS patients were functionally characterized (30). Further characterization of recombinant Factor B K323E showed that it is functionally very similar to a C3 nephritic factor, which most of the times does not allow activation of the complement terminal pathway (31). Two aHUS patients with another Factor B pathogenic variant and very low C3 levels in plasma have been recently described (32), and the authors consider that the excessive activation of the fluid-phase C3 convertase could predispose to aHUS or to C3 glomerulonephritis, depending on the presence of additional predisposing genes or environmental factors. In this context, it could be relevant that the aHUS-risk haplotype *MCPgggac* is present in our patient with the K323E mutation in Factor B, and in all the affected members of the family carrying the F286L mutation (4). One of the members of that family is a girl who presented HUS at the age of 3 months and had a bad clinical evolution, comparable in many aspects to the evolution of our patient with the K323E mutation. Nonetheless, C3 levels in the girl’s plasma were never as low as observed in our patient; this could reflect that the K323E mutation is more pathogenic for Factor B function than the F286L mutation. The girl with the F286L mutation received a kidney graft in 2011 and has remained stable without Eculizumab prophylaxis (5). It is likely that the absence of the *MCPggaac* aHUS-risk haplotype in the kidney graft have contributed to the successful evolution of this patient after transplantation. In the case of the patient with the K323E mutation, further genetic studies showed that donor and receptor shared the *MCPggaac* aHUS-risk haplotype but not the *CFH(H3)* haplotype; the absence of the *CFH*-risk polymorphism in addition to the presence of wild-type Factor B in the liver graft likely contributed to a better regulation of the C3 convertase in the receptor.

In conclusion, we describe the clinical evolution and therapeutic strategy in a boy with a gain–of-function mutation in complement Factor B. CLKT as a therapeutic option was justified because of a torpid evolution during 8 years, with several life-threatening events and the unavailability of complement-inhibiting drugs at that time. CLKT allowed the immediate restauration of kidney function and a stable evolution for four years, but after a B-cell lymphoma and a liver rejection, the patient needed a second liver graft 11 years later. Renal function and complement levels have remained normal all the time since the CKLT, and the patient has not suffered any aHUS recurrence.

## Data Availability Statement

The original contributions presented in the study are included in the article/**Supplementary Material**. Further inquiries can be directed to the corresponding author.

## Ethics Statement

The studies involving human participants were reviewed and approved by Comité de Ética del Hospital Universitario “La Paz”. Hospital La Paz. Madrid. Spain. Written informed consent to participate in this study was provided by the patient or their parents.

## Author Contributions

ÁA-M, MM-H, LE-R, and DL-B gathered clinical data. EG-F contributed to the analysis of the biopsy. PS-C, ÁA-M, MM-H, and ML-T prepared figures. ML-T compiled the data for the case-study, and wrote the first draft of the manuscript. SRC and PS-C revised the initial draft of the paper. All authors contributed to the article and approved the submitted version.

## Funding

ML-T, SRC, and PS-C are supported by the Spanish Autonomous Region of Madrid (Complement II-CM network; B2017/BMD-3673).

## Author Disclaimer

All claims expressed in this article are solely those of the authors and do not necessarily represent those of their affiliated organizations, or those of the publisher, the editors and the reviewers. Any product that may be evaluated in this article or claim that may be made by its manufacturer is not guaranteed or endorsed by the publisher.

## Conflict of Interest

The authors declare that the research was conducted in the absence of any commercial or financial relationships that could be construed as a potential conflict of interest

## Publisher’s Note

All claims expressed in this article are solely those of the authors and do not necessarily represent those of their affiliated organizations, or those of the publisher, the editors and the reviewers. Any product that may be evaluated in this article, or claim that may be made by its manufacturer, is not guaranteed or endorsed by the publisher.

## Supplementary Material

Supplementary Figure 1 Kidney biopsy. **(A)** Hematoxylin and Eosin staining of a glomerulus, showing mesangiolysis with fragmented red blood cells and double contours in capillary walls. **(B)** Masson trichrome staining, showing small arteries with intimal thickening, mucoid intimal edema, and endothelial swelling.

Supplementary Figure 2 Acrocyanosis on the feet fingers. The two images correspond to a microangiopathy episode occurring after the patient underwent bilateral nephrectomy.

## Abbreviations

aHUS, atypical Hemolytic Uremic syndrome; DGKE, Diacylglycerol kinase Epsilon; CLKT, combined liver kidney transplantation; DSA, donor specific antibodies; MFI, median fluorescence intensity.

## References

[1] Cody EM Dixon BP. Hemolytic Uremic Syndrome. Pediatr Clin North Am (2019) 66(1):235–46. doi: 10.1016/j.pcl.2018.09.011 30454746

[2] Nester CM Barbour T de Cordoba SR Dragon-Durey MA Fremeaux-Bacchi V Goodship TH. Atypical aHUS: State of the Art. Mol Immunol (2015) 67(1):31–42. doi: 10.1016/j.molimm.2015.03.246 25843230

[3] Saland JM Ruggenenti P Remuzzi G Consensus Study Group. Liver-Kidney Transplantation to Cure Atypical Hemolytic Uremic Syndrome. J Am Soc Nephrol (2009) 20(5):940–9. doi: 10.1681/ASN.2008080906 19092117

[4] Goicoechea de Jorge E Harris CL Esparza-Gordillo J Carreras L Arranz EA Garrido CA. Gain-Of-Function Mutations in Complement Factor B Are Associated With Atypical Hemolytic Uremic Syndrome. Proc Natl Acad Sci U S A (2007) 104(1):240–5. doi: 10.1073/pnas.0603420103 PMC1765442 17182750

[5] Sánchez-Moreno A de la Cerda F Rodríguez-Barba A Fijo J Bedoya R Arjona E. Is the Atypical Hemolytic Uremic Syndrome Risk Polymorphism in Membrane Cofactor Protein MCPggaac Relevant in Kidney Transplantation? A Case Report. Pediatr Transplant (2021) 25(3):e13903. doi: 10.1111/petr.13903 33217135

[6] Alonso Melgar A Melgosa M López-Trascasa M Navarro M de la Vega A and Sánchez-Corral P. Successful Combined Liver and Kidney Transplantation in a Child With Hypocomplementemic Atypical Haemolytic Uremic Syndrome (aHUS) Due to a Factor B Mutation. Pediatr Nephrol (2011) 26:1355 (Abstract). doi: 10.1007/s00467-011-1907-9

[7] Alper CA Rosen FS. Genetics of the Complement System. Adv Hum Genet (1976) 7:141–88. doi: 10.1007/978-1-4757-0659-8_4 827931

[8] Remuzzi G Ruggenenti P Codazzi D Noris M Caprioli J Locatelli G. Combined Kidney and Liver Transplantation for Familial Haemolytic Uraemic Syndrome. Lancet (2002) 359(9318):1671–2. doi: 10.1016/S0140-6736(02)08560-4 12020532

[9] Cheong HI Lee BS Kang HG Hahn H Suh KS Ha IS. Attempted Treatment of Factor H Deficiency by Liver Transplantation. Pediatr Nephrol (2004) 19(4):454–8. doi: 10.1007/s00467-003-1371-2 14986080

[10] Remuzzi G Ruggenenti P Colledan M Gridelli B Bertani A Bettinaglio P. Hemolytic Uremic Syndrome: A Fatal Outcome After Kidney and Liver Transplantation Performed to Correct Factor H Gene Mutation. Am J Transplant (2005) 5(5):1146–50. doi: 10.1111/j.1600-6143.2005.00783.x 15816899

[11] Saland JM Emre SH Shneider BL Benchimol C Ames S Bromberg JS. Favorable Long-Term Outcome After Liver-Kidney Transplant for Recurrent Hemolytic Uremic Syndrome Associated With a Factor H Mutation. Am J Transplant (2006) 6(8):1948–52. doi: 10.1111/j.1600-6143.2006.01375.x 16889549

[12] Jalanko H Peltonen S Koskinen A Puntila J Isoniemi H Holmberg C. Successful Liver-Kidney Transplantation in Two Children With aHUS Caused by a Mutation in Complement Factor H. Am J Transplant (2008) 8(1):216–21. doi: 10.1111/j.1600-6143.2007.02029.x 17973958

[13] Saland JM Shneider BL Bromberg JS Shi PA Ward SC Magid MS. Successful Split Liver-Kidney Transplant for Factor H Associated Hemolytic Uremic Syndrome. Clin J Am Soc Nephrol (2009) 4(1):201–6. doi: 10.2215/CJN.02170508 PMC2615708 19005013

[14] Opelz G Margreiter R Döhler B. Prolongation of Long-Term Kidney Graft Survival by a Simultaneous Liver Transplant: The Liver Does it, and the Heart Does it Too. Transplantation (2002) 74(10):1390–4; discussion 1370-1. doi: 10.1097/00007890-200211270-00008 12451237

[15] Ranawaka R Lloyd C McKiernan PJ Hulton SA Sharif K Milford DV. Combined Liver and Kidney Transplantation in Children: Analysis of Renal Graft Outcome. Pediatr Nephrol (2016) 31(9):1539–43. doi: 10.1007/s00467-016-3396-3 27105881

[16] Creput C Durrbach A Samuel D Eschwege P Amor M Kriaa F. Incidence of Renal and Liver Rejection and Patient Survival Rate Following Combined Liver and Kidney Transplantation. Am J Transplant (2003) 3(3):348–56. doi: 10.1034/j.1600-6143.2003.00050.x 12614293

[17] Debray D Baudouin V Lacaille F Charbit M Rivet C Harambat J. Pediatric Transplantation Working Group of the French Speaking Society of Transplantation. *De Novo* Malignancy After Solid Organ Transplantation in Children. Transplant Proc (2009) 41(2):674–5. doi: 10.1016/j.transproceed.2008.12.020 19328954

[18] Calne RY Davis DR Hadjiyannakis E Sells RA White D Herbertson BM. Immunosuppressive Effects of Soluble Cell Membrane Fractions, Donor Blood and Serum on Renal Allograft Survival. Nature (1970) 227(5261):903–6. doi: 10.1038/227903a0 4988655

[19] Knechtle SJ Kwun J. Unique Aspects of Rejection and Tolerance in Liver Transplantation. Semin Liver Dis (2009) 29(1):91–101. doi: 10.1055/s-0029-1192058 19235662

[20] Mohan S Palanisamy A Tsapepas D Tanriover B Crew RJ Dube G. Donor-Specific Antibodies Adversely Affect Kidney Allograft Outcomes. J Am Soc Nephrol (2012) 23(12):2061–71. doi: 10.1681/ASN.2012070664 PMC3507372 23160511

[21] Everly MJ Rebellato LM Haisch CE Ozawa M Parker K Briley KP. Incidence and Impact of *De Novo* Donor-Specific Alloantibody in Primary Renal Allografts. Transplantation (2013) 95(3):410–7. doi: 10.1097/TP.0b013e31827d62e3 23380861

[22] Taner T Heimbach JK Rosen CB Nyberg SL Park WD Stegall MD. Decreased Chronic Cellular and Antibody-Mediated Injury in the Kidney Following Simultaneous Liver-Kidney Transplantation. Kidney Int (2016) 89(4):909–17. doi: 10.1016/j.kint.2015.10.016 26924059

[23] Del Bello A Thaunat O Le Quintrec M Bestard O Durrbach A Perrin P. Combined Liver-Kidney Transplantation With Preformed Anti-Human Leukocyte Antigen Donor-Specific Antibodies. Kidney Int Rep (2020) 5(12):2202–11. doi: 10.1016/j.ekir.2020.09.018 PMC7710847 33305113

[24] de Castro C Grossi F Weitz IC Maciejewski J Sharma V Roman E. C3 Inhibition With Pegcetacoplan in Subjects With Paroxysmal Nocturnal Hemoglobinuria Treated With Eculizumab. Am J Hematol (2020) 95(11):1334–43. doi: 10.1002/ajh.25960 PMC7693064 33464651

[25] Hillmen P Szer J Weitz I Röth A Höchsmann B Panse J. Pegcetacoplan Versus Eculizumab in Paroxysmal Nocturnal Hemoglobinuria. N Engl J Med (2021) 384(11):1028–37. doi: 10.1056/NEJMoa2029073 33730455

[26] Hoy SM. Pegcetacoplan: First Approval. Drugs (2021) 81(12):1423–30. doi: 10.1007/s40265-021-01560-8 34342834

[27] Bacchetta J Mekahli D Rivet C Demède D Leclerc AL. Pediatric Combined Liver-Kidney Transplantation: A 2015 Update. Curr Opin Organ Transplant (2015) 20(5):543–9. doi: 10.1097/MOT.0000000000000225 26270957

[28] Zuber J Le Quintrec M Morris H Frémeaux-Bacchi V Loirat C Legendre C. Targeted Strategies in the Prevention and Management of Atypical HUS Recurrence After Kidney Transplantation. Transplant Rev (Orlando) (2013) 27(4):117–25. doi: 10.1016/j.trre.2013.07.003 23937869

[29] Saland J. Liver-Kidney Transplantation to Cure Atypical HUS: Still an Option Post-Eculizumab? Pediatr Nephrol (2014) 29(3):329–32. doi: 10.1007/s00467-013-2722-2 24362724

[30] Marinozzi MC Vergoz L Rybkine T Ngo S Bettoni S Pashov A. Complement Factor B Mutations in Atypical Hemolytic Uremic Syndrome-Disease-Relevant or Benign? J Am Soc Nephrol (2014) 25(9):2053–65. doi: 10.1681/ASN.2013070796 PMC4147975 24652797

[31] Urban A Borowska A Felberg A van den Heuvel L Stasiłojć G Volokhina E. Gain of Function Mutant of Complement Factor B K323E Mimics Pathogenic C3NeF Autoantibodies in Convertase Assays. Autoimmunity (2018) 51(1)18–24. doi: 10.1080/08916934.2017.1423286 29308663

[32] Zhang Y Kremsdorf RA Sperati CJ Henriksen KJ Mori M Goodfellow RX. Mutation of Complement Factor B Causing Massive Fluid-Phase Dysregulation of the Alternative Complement Pathway can Result in Atypical Hemolytic Uremic Syndrome. Kidney Int (2020) 98(5):1265–74. doi: 10.1016/j.kint.2020.05.028 PMC7606633 32540405

