# Supplementary figures and images for: Case Report: Combined Liver-Kidney Transplantation to Correct a Mutation in Complement Factor B in an Atypical Hemolytic Uremic Syndrome Patient
Source: Front Immunol. 2021 Oct 14;12:751093. doi: 10.3389/fimmu.2021.751093 (PMC8551365; doi:10.3389/fimmu.2021.751093)

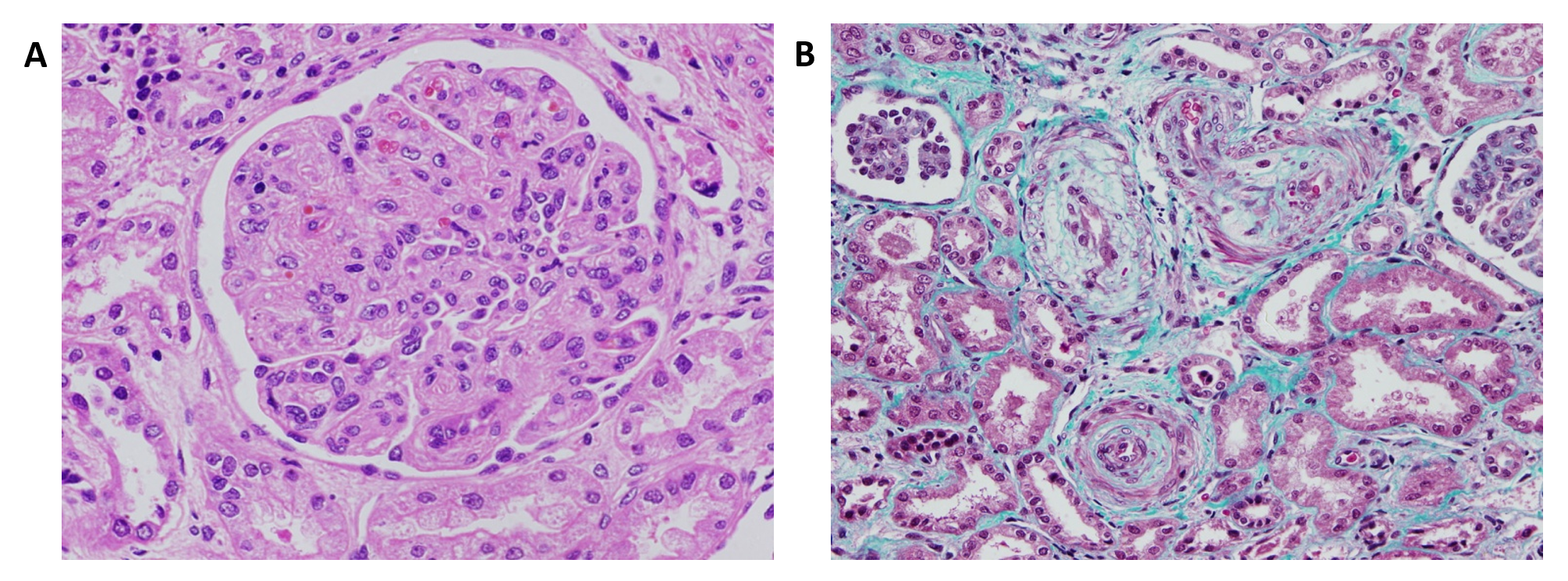

Supplement: Supplementary Figure 1 — Kidney biopsy. (A) Hematoxylin and Eosin staining of a glomerulus, showing mesangiolysis with fragmented red blood cells and double contours in capillary walls. (B) Masson trichrome staining, showing small arteries with intimal thickening, mucoid intimal edema, and endothelial swelling. [file Image_1.tif]

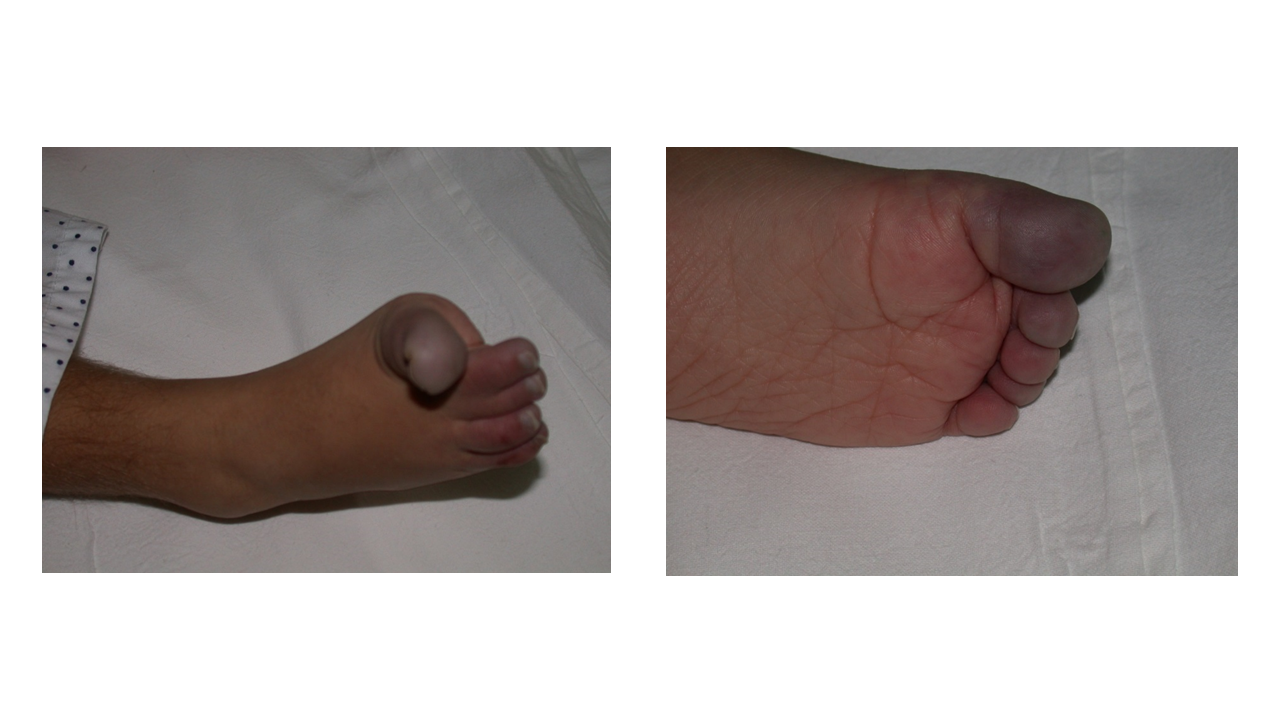

Supplement: Supplementary Figure 2 — Acrocyanosis on the feet fingers. The two images correspond to a microangiopathy episode occurring after the patient underwent bilateral nephrectomy. [file Image_2.tif]
